# *Aspergillus oryzae* accelerates the conversion of ergosterol to ergosterol peroxide by efficiently utilizing cholesterol

**DOI:** 10.3389/fgene.2022.984343

**Published:** 2022-08-22

**Authors:** Shangkun Qiu, Qicong Liu, Ya Yuan, Hong Zhou, Bin Zeng

**Affiliations:** ^1^ College of Pharmacy, Shenzhen Technology University, Shenzhen, China; ^2^ Jiangxi Province Center for Disease Control and Prevention, Nanchang, China

**Keywords:** *Aspergillus oryzae*, utilize, cholesterol, Osh3, ergosterol peroxide

## Abstract

It is well-known that excessive cholesterol leads to hypercholesterolemia, arteriosclerosis, coronary heart disease, stroke, and other diseases, which seriously threatens human health. *Lactobacillus*, a prokaryote, is reported to utilize cholesterol in the environment. However, little research focuses on the cholesterol utilization by eukaryote. Hence, the objectives of the present study were to investigate the mechanism of cholesterol utilization by the eukaryote and determine the role of oxysterol binding protein in this process. Our results showed for the first time that *Aspergillus oryzae*, a food-safe filamentous fungus, can utilize cholesterol efficiently. Our results also demonstrated that cholesterol utilization by *A. oryzae* might promote the conversion of ergosterol to ergosterol peroxide. Osh3, an oxysterol binding protein, can bind sterols (e.g., cholesterol, ergosterol, and ergosterol peroxide) and plays an important role in sterols transportation. This research is of considerable significance for developing low-fat food and cholesterol-lowering probiotics.

## Introduction

Sterols are found in most organisms. Eukaryotes synthesize three main types of sterols: cholesterol in vertebrates, phytosterols (e.g., campesterol, stigmasterol, and sitosterol) in plants, and ergosterol in fungi (Dupont et al., 2012). Cholesterol, an important sterol, is an essential component of the human body, accounting for 0.2% of the total weight of the human body. Cholesterol has three main physiological functions: formation of cholic acid and the cell membrane, synthesis of hormones (Yu and Cui, 2001). Excessive cholesterol leads to hypercholesterolemia, arteriosclerosis, coronary heart disease, stroke, and other diseases, which seriously threaten human health (Steinberg, 2006). Therefore, reducing the cholesterol level in the human body is being actively investigated to prevent diseases caused by high cholesterol content. Current studies on cholesterol reduction have mainly focused on microbial degradation of cholesterol, which has many advantages. The absorption of cholesterol by microorganisms from nature is a strict “efficiency” choice of biological evolution, which has the advantages of strong specificity, high efficiency, and few side effects (Niu, 2000). At present, studies on cholesterol degradation by microorganisms focus mainly on the use of *Lactobacillus*, a prokaryote, although few groups are investigating cholesterol degradation by fungi, as eukaryotes are more complex than prokaryotes (Damodharan et al., 2016; Ding et al., 2017).

*A. oryzae* is a filamentous fungus widely used to produce fermented food. *A. oryzae* does not produce mycotoxins but can produce various enzymes with biological activities such as protease, peptidase, and amylase during soy sauce brewing. This will increase soy sauce’s nutritional value, flavor, and taste, so it is considered a safe food-grade fermentation strain (Sugiyama, 1984;Bechman et al., 2012). *A. oryzae* harbors five oxysterol-binding protein-related protein (ORP) family proteins, namely oxysterol binding proteins homologous 1 (Osh1), Osh3, Osh5, Osh7, and ORP8, which play essential roles in lipid metabolism and sterol transportation of *A. oryzae* (Ma et al., 2019). Therefore, understanding the mechanism of cholesterol degradation by *A. oryzae* is important.

Oxysterol-binding protein was first identified in mammals and was defined as oxysterol-binding protein (OSBP) in 1985, followed by the identification of ORP in mammals and Osh in yeast (Taylor and Kandutsch, 1985). As essential non-vesicular transport proteins, OSBP and ORP play important roles in cell growth and metabolism. Especially at membrane contact sites (MCS) formed by the endoplasmic reticulum (ER) and other organelles (e.g., Golgi body, mitochondria, cell membrane, and lysosomes) (Du et al., 2015). OSBP and ORPs bind various sterols (e.g., 25-hydroxycholesterol, 22-hydroxycholesterol, cholesterol, ergosterol) *via* the oxysterol-binding protein-related domain (ORD) and bind phospholipids *via* their pleckstrin homology (PH) domain (Kentala et al., 2015; Ma et al., 2019). OSBP and ORP transport phospholipids formed on the plasma membrane or other organelles membranes to ER, which are hydrolyzed by Sac1 phosphatase enzyme, and reverse transport sterols from the ER to the corresponding membranes for metabolic activities (Mesmin et al., 2017).

In this study, we cultured *A. oryzae* in cholesterol medium and determined the utilization properties of cholesterol by *A. oryzae*. At the same time, we also measured the other sterols (e.g., ergosterol, ergosterol peroxide) content in *A. oryzae*. The function of Osh3 was also investigated by the microscale thermophoresis (MST) assay to determine its important roles in lipid metabolism and sterols transport. These results may be crucial for developing low-fat food and cholesterol-lowering probiotics.

## Materials and methods

### Materials

*Escherichia. Coli Transetta* DE3 cells were purchased from Takara Biomedical Technology (Beijing) Co., Ltd. The wild type strains of *A. oryzae* 3.042 (CICC 40092) were preserved in JiangXi Province Key Laboratory Bioprocess Engineering. His-Ni gravity columns were purchased from Takara Biomedical Technology (Beijing) Co., Ltd. Protein Purification Kits was purchased from Beijing ComWin Biotech Co.,Ltd. Stigmasterol, β-sitosterol, campesterol, and cholesterol standards were all purchased from ChemFaces Biotechnology (Wuhan) Co., Ltd. Ergosterol standard was purchased from Sigma Aldrich (Shanghai) Trading Co., Ltd. 7-Keto-cholesterol and 25-hydroxycholesterol standards were purchased from Santa Cruz Biotechnology (United States) Inc. Bincinchoninic acid (BCA) protein quantitation kit was purchased from Tiangen Biochemical Technology (Beijing) Co., Ltd. Ergosterol peroxide and cerevisterol standards were purchased from Kmaels (Shanghai) Biotechnology Co., Ltd. Cholesterol with purity of 99% was purchased from Beijing Biotopped Technology Co., Ltd. Protein labeling kit and capillaries were purchased from NanoTemper Technologies (Beijing).

### Mediums and primary solvents

Cholesterol CD medium was made by the Czapek-Dox (CD) medium sterilized at 115°C for 15 min (Li et al., 2021). Depending on the concentration of cholesterol medium to be made, the appropriate amount of cholesterol (99%) was sterilized using ultraviolet radiation for 30–40 min, added to the above sterile CD medium and shaken well. The saponification solution was prepared by adding 25 g potassium hydroxide in 35 ml ddH_2_O for dissolution, followed by the addition of absolute alcohol to a volume of 100 ml; the solution was shaken well and refrigerated at 4°C (Wang et al., 2018). The methods of making the binding buffer, lysis buffer, and elution buffer were modified from Protein Purification Kits and described in Supplementary information (Ma et al., 2019).

### *A. oryzae* 3.042 fermentation in normal glucose CD medium and cholesterol medium

The spore suspension of *A. oryzae* 3.042 was added to the normal CD medium and cholesterol CD medium. 200 μl of *A. oryzae* spore suspension was added to every 100 ml medium and placed in a shaker moving at 160 rpm at 30°C for fermentation. There were five different investigations: 1) Three experiments were performed simultaneously for culturing *A. oryzae* in the normal CD mediums: the three experiments were fermented for 48, 72, and 96 h, respectively. 2) Three experiments were performed simultaneously for cholesterol medium fermentation: The three experiments were fermented for 48, 72, and 96 h, respectively. 3) When the cholesterol medium was fermented for 48 h, the same amount of cholesterol was supplemented in the medium again. Two experiments were performed simultaneously: These two experiments were fermented for 72 h and 96 h, respectively. 4) The same amount of cholesterol was added again when the normal CD medium was fermented for 48 h. Two experiments were performed simultaneously: The two experiments were fermented for 72 h and 96 h, respectively. 5) *A. oryzae* was fermented for 48, 72, and 96 h in the normal medium at the same time: the first experiment was fermented for 48 h, then *A. oryzae* was killed in the sterilizer at 121°C for 20 min. The dead *A. oryzae* was treated with ddH_2_O for cleaning. Then it was added to the cholesterol medium and cultured at 30°C for 48 h to assess cholesterol adsorption by *A. oryzae* after culturing for 48 h. The second and third experiment were fermented for 72 h and 96 h respectively. Then *A. oryzae* was killed and the dead *A. oryzae* was treated with ddH_2_O for cleaning. It was added to the cholesterol medium and cultured at 30°C for 72 h and 96 h to assess cholesterol adsorption by *A. oryzae* after culturing for 72 h and 96 h (Zhou et al., 2017). Three replicates were included for each experiment.

### Mycelium treatment of *A. oryzae* 3.042

The mycelium of *A. oryzae* was cleaned with ddH_2_O, dried in an oven at 60°C, and ground in a mortar. The powder weighting about 0.0200 g was placed in a 50 ml centrifuge tube, followed by the addition of a 3 ml saponification solution, vortexing and shaking for 1 min, and incubation in an 85°C water bath for 1 h. After saponification, the mixture was cooled to room temperature, followed by adding 1 ml ddH_2_O and 3 ml n-heptane and vortexing for 3 min (Wang et al., 2018). The mixture was incubated at room temperature for 15 min, after which 2 ml of the n-heptane layer was aspirated and stored at −20°C for 24 h. Before the detection, 10–200 μl n-heptane extraction layer was drawn and diluted to 1 ml with methanol, centrifuged at 12,000 g for 1 min, and filtered through 0.22 μm membrane, followed by high-performance liquid chromatography (HPLC).

### Determination of cholesterol and peroxide ergosterol in mycelium

The detection conditions of cholesterol and peroxide ergosterol were identical. The standard samples were detected using the HPLC system of Waters, and the standard curve was plotted. A C18 reverse chromatographic column was operated at 38°C. The mobile phase was chromatography-grade pure methanol at a 1 ml/min flow rate. The injection volume was 20 μl, and the detection time for each sample was 20 min. The samples were detected using a UV detector at 205 nm. Before detection, the samples was centrifuged for 1 min at 12,000 g and filtered with a 0.22 μm filter membrane.

### Determination of ergosterol in mycelium

The standard samples were detected using Waters’ HPLC system, and the standard curve was plotted. The mobile phase was methanol and water in a 95:5 ratio. The column used was a C_18_ reverse column, column temperature was 30°C, the flow rate was 1.5 ml/min, injection volume was 20 μl, and the time required for detection of each sample was 20 min. A UV detector was used at 282 nm. Before detection, the samples were centrifuged for 1 min at 12,000 g and filtered through a 0.22 μm filter membrane.

### Statistical analysis

Comparisons of statistically significant differences between two groups were made by Student’s *t*-tests. Graphs were prepared in Graphpad Prism (version 7.00).

### Sequence and structural analysis of Osh proteins

The sequences of the genes encoding the Osh proteins in *A. oryzae* 3.042 were identified by comparing five *Osh* gene sequences of *A. nidulans* with the whole genome sequence of *A. oryzae* 3.042 using NCBI’s BLAST program (Bühler et al., 2015). The conserved domains were analyzed using the SMART program (http://smart.emblheidelberg.de/).

### Synthesis of Osh3 and Osh3 expression

The accession number for the nucleotide sequence of *Osh3* is XM_001824384.3 in the NCBI database. Six His tags were added to the 5′ end (N-terminus) of *Osh3*. *Osh3* was synthesized by GenScript (Nanjing) Co., Ltd. and connected to the pET28 vector.

Osh3 was expressed in *E. coli Transseta* DE3 using isopropyl β-d-1-thiogalactopyranoside (IPTG) induction. Osh3-expressing colonies were inoculated in 10 ml LB medium supplemented with the appropriate antibiotic and incubated at 37°C with shaking at 180 rpm. The next day, 2 ml culture was taken and diluted in 200 ml fresh medium and allowed to grow for 2 h to OD600 of 0.4–0.6, after which IPTG was added to a final concentration of 0.5 mM. The induction time for Osh3 was 10 h at 27°C, after which the cells were pelleted and stored at −80°C until further processing (Ma et al., 2019).

### Purification and quantification of Osh3

The purification process was modified from the instruction of Protein Purification Kits. The cell pellets were obtained by centrifugation at 10,000 *g* for 10 min and resuspended in 10 ml lysis buffer, followed by lysis *via* sonication. The supernatant was injected into a His-Ni-agarose resin column. Then the target protein was eluted by elution buffer with gradient of imidazole (50 mM, 100 mM, and 250 mM). The size and purity of the Osh3 were verified by SDS-PAGE. The fractions containing high concentrations of pure protein were mixed with glycerol to a final volume of 50% glycerol and stored at −80°C (Ma et al., 2019). The protein quantification was based on the BCA kit. Protein concentration was determined according to the absorbance at 562 nm of the eluates.

### Fluorescent labeling of proteins and binding experiments with ligands

Fluorescent labeling of proteins was based on that of the instruction of Protein labeling kit. The binding assay was performed using the MST method with a NanoTemper monolith NT.115. The A-column was centrifuged at 1,500 g at 4°C for 1 min to remove the 20% ethanol protective solution. Next, the particles in the labeling buffer were dissolved in 3 ml ddH_2_O, from which 250 μl was added to the A column and cleaned twice. Then, 100 μl protein solution was added to column A and centrifuged at 4°C for 1 min at 1,500 g; the protein solution was collected in a 1.5 ml centrifuge tube. The column was cleaned twice with 300 μl 1 × PBS, followed by the addition of 20% ethanol to column A, and storage at 4°C. The collected 100 μl protein solution was mixed with 95 μl labeling buffer, followed by the addition of 5 μl fluorescent dye (the fluorescent dye was dissolved with 30 μl DMSO) in a total volume of 200 μl; the solution was incubated for 30 min in dark at room temperature. In the last 10 min, the B-column was cleaned with binding buffer. Then, 200 μl of the fluorescent-labeled protein solution was added to B-column; 300 μl of binding buffer was added to B-column; and 600 μl of this binding buffer was added to column B. After removing the first two drops, the remaining liquid was collected in a 1.5 ml centrifuge tube, with one centrifuge tube for every 200 μl eluate. The column was washed with 1× PBS and sealed with 20% ethanol at 4°C; the labeled protein was used immediately or stored at −20°C for future use.

The above labeled fluorescent protein was centrifuged at 2000 g for 5 min, mixed with ligands, and tested using the NanoTemper monolith NT.115. A capillary was used to aspirate the labeled protein. The LED power was set to 20% to detect the fluorescence, and the protein concentration was adjusted according to the detected fluorescence value to obtain fluorescence value between 400–2000. The concentration finder software was used to analyze the optimal concentration of ligands. According to the analysis results, 1 × PBS was used to dilute the ligands. 10 μl of 1 × PBS was taken; the first 200 μl centrifuge tube was not added, each of the other 15 centrifuge tubes needs to be added with 10 μl 1 × PBS. 10 μl of the ligand was added to the first centrifuge tube, and 10 μl ligand was again added to the second centrifuge tube and mixed, after which 10 μl solution was aspirated from the second centrifuge tube and added to the third centrifuge tube. This is a serial dilution of the ligand. This process was repeated until the 16th centrifuge tube, and the last 10 μl was discarded. 10 μl fluorescent-labeled protein solution was added to all sixteen centrifuge tubes separately, mixed well, and incubated at room temperature for 5 min. Then, 10 μl of liquid was aspirated using sixteen capillaries and tested on the NanoTemper monolith NT.115. The results were analyzed using the MO. Mffinity analysis (× 86) software (Ma et al., 2019).

## Results

### Utilization properties of cholesterol by *A. oryzae*

**FIGURE 1:**
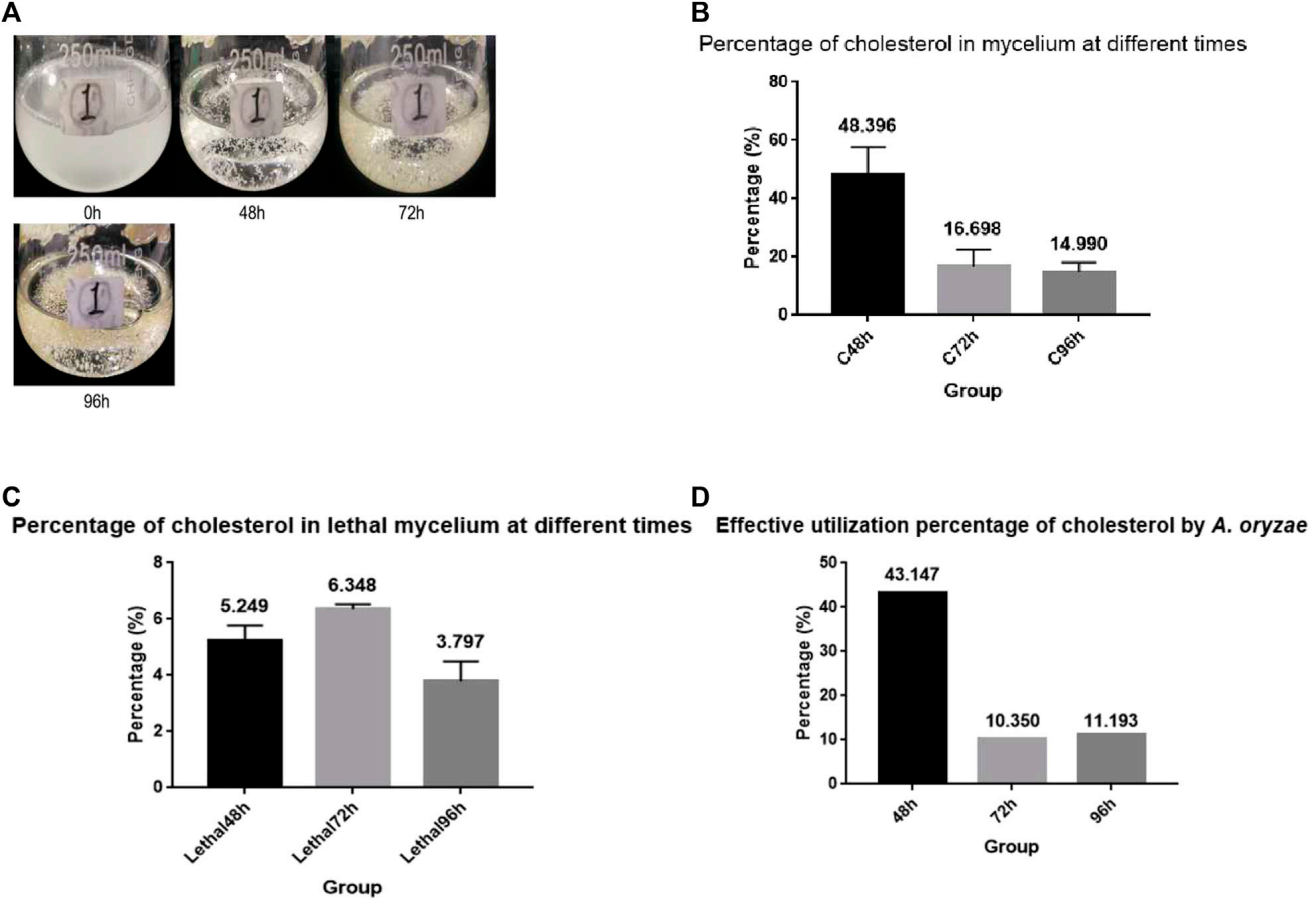
*A. oryzae* can utilize cholesterol. **(A)** Cholesterol medium was fermented by *A. oryzae*, and the growth condition of *A. oryzae* in different periods of fermentation were determined. **(B)** The percentage of cholesterol in mycelium dry weight at different time points. **(C)** The percentage of cholesterol in lethal mycelium dry weight at different time points. **(D)** Effective absorption percentage of cholesterol by *A. oryzae*. Three independent experiments were performed.

To investigate whether *A. oryzae* can absorb and utilize cholesterol from the environment, we prepared CD culture medium with cholesterol as the carbon source, and the spore suspension of *A. oryzae* was inoculated for fermentation. We observed that the carbon source does not have to be dissolved in the culture medium to be absorbed by *A. oryzae*. When 0.5 g cholesterol was added to 100 ml CD medium and shaken sufficiently, cholesterol was evenly distributed in the medium to form a turbid liquid. *A. oryzae* can grow rapidly in the medium with cholesterol as the carbon source, and the medium clarified with time (Figure 1A). Therefore, we concluded that *A. oryzae* can absorb and utilize the cholesterol in the medium. After the fermentation, the cholesterol content in the mycelium was determined using HPLC. The results showed the presence of cholesterol in the mycelium of *A. oryzae*, and the percentage of cholesterol in mycelium dry weight decreased with time (Figure 1B). The mycelium’s cholesterol percentage was 48.396% when *A. oryzae* was cultured for 48 h, whereas it was 16.698% when cultured for 72 h. The mycelium’s cholesterol percentage decreased to 14.990% at 96 h. This confirmed that *A. oryzae* could absorb and utilize cholesterol, and the absorption percentage of cholesterol by *A. oryzae* was maximum at 48 h. To eliminate the interference of *A. oryzae* mycelium on physisorption of cholesterol, we cultured *A. oryzae* in normal medium with glucose as the carbon source, and killed the mycelium at 121°C when *A. oryzae* was cultured for 48, 72, and 96 h. The dead *A. oryzae* was added to the cholesterol medium and cultured for 48, 72, and 96 h. The content of cholesterol in the mycelium was determined using HPLC. The results showed that the dead mycelium of *A. oryzae* showed physisorption of cholesterol, although the content of the adsorbed cholesterol was low. The percentage of cholesterol in the dead mycelium dry weight at 48, 72, and 96 h was 5.249%, 6.348%, and 3.797%, respectively, indicating that *A. oryzae* can absorb and utilize cholesterol. The effective absorption percentages were 43.147%, 10.350%, and 11.193% at 48, 72, and 96 h, respectively (Figures 1C,D).

**FIGURE 2:**
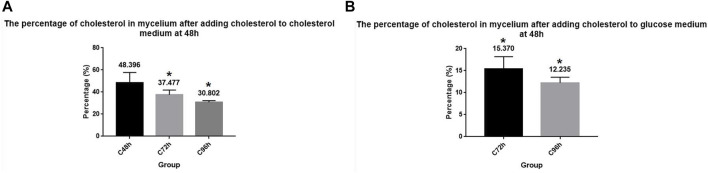
*A. oryzae* can absorb and utilize cholesterol instantaneously. **(A)** After fermentation of cholesterol medium by *A. oryzae* for 48 h, the same amount of cholesterol was added to the medium again, and the percentage of cholesterol in mycelium dry weight at different time points was estimated. **(B)** After fermentation of glucose medium by *A. oryzae* for 48 h, the same amount of cholesterol was added to the medium again, and the percentage of cholesterol in mycelium dry weight at different time points was determined. Three independent experiments were performed. The asterisk denotes statistically significant differences between the **(A)** and **(B)** at 72 h or 96 h based on Student’s *t*-tests (*p < 0.05).

To investigate whether *A. oryzae* can absorb and utilize cholesterol instantaneously, the same amount of cholesterol was added to the cholesterol medium when it was cultured for 48 h (C + C culture medium), and the cholesterol content in the mycelium was measured when it was cultured for 72 h and 96 h. The results showed that the cholesterol content was 37.477% and 30.802% at 72 h and 96 h, respectively (Figure 2A). The cholesterol content of the mycelium increased by 20.779% and 16.609% at 72 h and 96 h, respectively, compared to that in the cholesterol medium without the addition of cholesterol at 48 h (C+ culture medium), although the cholesterol content still decreased with time. This suggested that cholesterol absorption by *A. oryzae* increased when cholesterol was again added to the medium. To eliminate the effect of cholesterol accumulated in the mycelium before 48 h, we cultured *A. oryzae* in glucose medium, added cholesterol to the medium at 48 h (G + C culture medium), and determined the cholesterol content in mycelium using HPLC. The results showed that the cholesterol content in mycelium dry weight was 15.370% and 12.235% at 72 h and 96 h, respectively, which decreased with time (Figure 2B). This indicated that *A. oryzae* could absorb and utilize cholesterol instantaneously.

### Cholesterol utilization by *A. oryzae* was essential for promoting the synthesis of ergosterol peroxide

**FIGURE 3:**
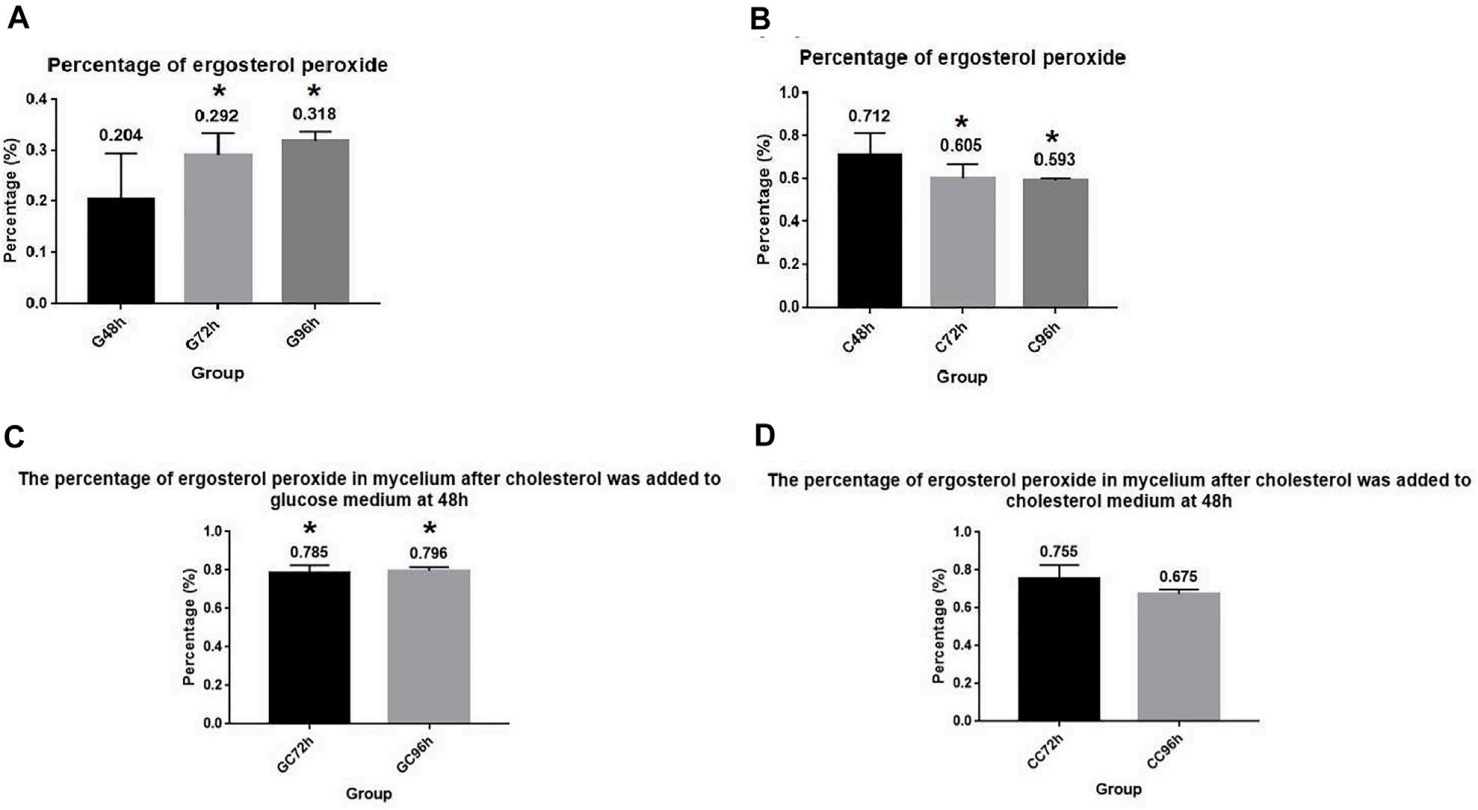
*A. oryzae* can promote the synthesis of ergosterol peroxide by utilizing cholesterol. **(A)** Normal glucose medium fermented by *A. oryzae*, and the percentage of ergosterol peroxide in mycelium dry weight at 48, 72, and 96 h was determined. **(B)** Cholesterol medium was fermented by *A. oryzae*, and the percentage of ergosterol peroxide in mycelium dry weight at 48, 72, and 96 h was determined. **(C)** Normal glucose medium was fermented by *A. oryzae*, and cholesterol was added to the medium again at 48 h. The percentage of ergosterol peroxide in the mycelium dry weight at 72 h and 96 h. **(D)** Cholesterol medium was fermented by *A. oryzae*, and the same amount of cholesterol was added to the medium again at 48 h. The percentage of ergosterol peroxide in the mycelium dry weight at 72 h and 96 h. Three independent experiments were performed. The asterisk denotes statistically significant differences between the **(A)** and **(B)**, and **(A)** and **(C)** at 72 h or 96 h based on Student’s *t*-tests (*p < 0.05).

Measurement of the ergosterol peroxide content revealed that *A. oryzae* could synthesize ergosterol peroxide, which was promoted by cholesterol utilization. The content of ergosterol peroxide in the mycelium cultured in the glucose medium (G+ culture medium) was determined using HPLC. We observed that the content of ergosterol peroxide in *A. oryzae* increased with time. The percentage of ergosterol peroxide in the mycelium dry weight was 0.204%, 0.292%, and 0.318% at 48, 72, and 96 h, respectively (Figure 3A). When we cultured *A. oryzae* in C+ culture medium, ergosterol peroxide was also detected in the mycelium at 48, 72, and 96 h, indicating that *A. oryzae* can also synthesized ergosterol peroxide in the cholesterol medium. The content of ergosterol peroxide in mycelium dry weight was 0.712%, 0.605%, and 0.593% at 48, 72, and 96 h, respectively, which was 0.508%, 0.313%, and 0.275% higher than that in glucose medium. This suggested that cholesterol utilization by *A. oryzae* can promote the synthesis of ergosterol peroxide, and the amount of ergosterol peroxide was highest at 48 h (Figure 3B).

When *A. oryzae* was cultured in G + C culture medium. The changes in ergosterol peroxide content in the mycelium were measured at 72 h and 96 h. The results showed that the content of ergosterol peroxide increased considerably compared to the ergosterol peroxide content in the G+ culture medium, and its percentage in mycelium dry weight was 0.785% and 0.796% at 72 h and 96 h, respectively. This indicated that *A. oryzae* can rapidly change the rate of ergosterol peroxide synthesis with the change in carbon sources in the environment, and that instantaneous utilization of cholesterol can promote the rapid synthesis of ergosterol peroxide (Figure 3C). To further investigate the effect of cholesterol utilization by *A. oryzae* on the synthesis of ergosterol peroxide, we cultured *A. oryzae* in C + C culture medium and measured the content of ergosterol peroxide in mycelium again at 72 h and 96 h. The results showed that the content of ergosterol peroxide in the mycelium was 0.755% and 0.675% at 72 h and 96 h, respectively, and that ergosterol peroxide content at 72 h was 0.150% higher than that in C+ culture medium. This suggested that cholesterol supplementation can accelerate the synthesis of ergosterol peroxide by *A. oryzae* and compensate for the decrease in ergosterol peroxide content over time (Figure 3D).

### Cholesterol utilization by *A. oryzae* will affect the intracellular ergosterol content

**FIGURE 4:**
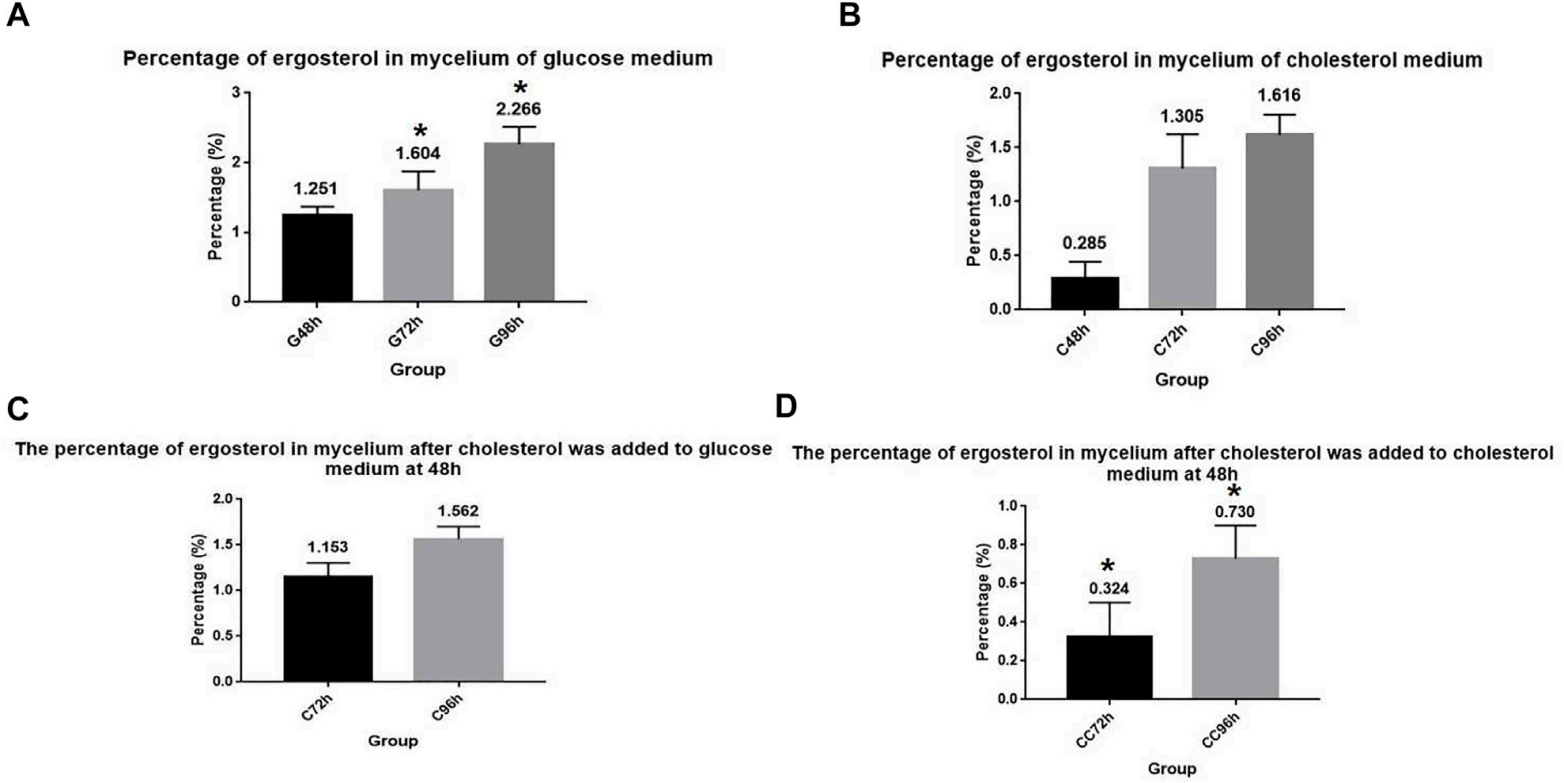
Cholesterol utilization by *A. oryzae* can reduce ergosterol content in cells. **(A)** Glucose medium was fermented by *A. oryzae,* and the percentage of ergosterol in mycelium dry weight at 48, 72, and 96 h was determined. **(B)** Cholesterol medium was fermented by *A. oryzae,* and the percentage of ergosterol in mycelium dry weight at 48, 72, and 96 h was determined. **(C)** Normal glucose medium was fermented by *A. oryzae* and cholesterol was added to the medium again at 48 h. The percentage of ergosterol in the mycelium dry weight at 72 h and 96 h was determined. **(D)** Cholesterol medium was fermented by *A. oryzae* and cholesterol was added to the medium again at 48 h. The percentage of ergosterol in the mycelium dry weight at 72 h and 96 h was determined. Three independent experiments were performed. The asterisk denotes statistically significant differences between the **(A)** and **(D)** at 72 h or 96 h based on Student’s *t*-tests (*p < 0.05).

Ergosterol is an essential synthetic substance in *A. oryzae* and an important component of the cell membrane (Solanko et al., 2018). We cultured *A. oryzae* in G+ culture medium and determined the ergosterol content in the mycelium. Results showed that the ergosterol content in *A. oryzae* mycelium increased with time, indicating that the synthesis of ergosterol increased with time (Figure 4A). The percentage of ergosterol in mycelium dry weight at 48, 72, and 96 h was 1.251%, 1.604%, and 2.266%, respectively. When *A. oryzae* was cultured in C+ culture medium and ergosterol content in mycelium in different time periods was determined, we found that ergosterol content in mycelium increased with time, indicating that ergosterol synthesis in *A. oryzae* increased with time (Figure 4B). However, the ergosterol content in mycelium cultured in C+ culture medium was lower than that in mycelium cultured in G+ culture medium. The percentage of ergosterol in mycelium dry weight was 0.285%, 1.305%, and 1.616% at 48, 72, and 96 h, respectively, indicating that *A. oryzae* can decrease ergosterol content *in vivo* when it utilized cholesterol as the carbon source. To further investigate the effect of cholesterol utilization by *A. oryzae* on ergosterol content in the mycelium, we cultured *A. oryzae* in G + C culture medium and C + C culture medium for 96 h at the end of which the ergosterol content in mycelium was detected using HPLC. The results showed that when *A. oryzae* was cultured in G + C culture medium, the ergosterol content in mycelium increased with time (Figure 4C). At 72 h and 96 h, the ergosterol content in the mycelium was 1.153% and 1.562%, respectively, which was 0.451% and 0.465% lower than that in G+ culture medium. The ergosterol level in *A. oryzae* mycelium decreased significantly after adding cholesterol in the cholesterol medium at 48 h (Figure 4D). The content of ergosterol was 0.324% and 0.730% at 72 h and 96 h, respectively, which was 0.981% and 0.886% lower than that in C+ culture medium. This indicated that the ergosterol content in the cells was significantly reduced when cholesterol was added to the culture medium.

### Sequence and structural analysis of osh proteins

**FIGURE 5:**
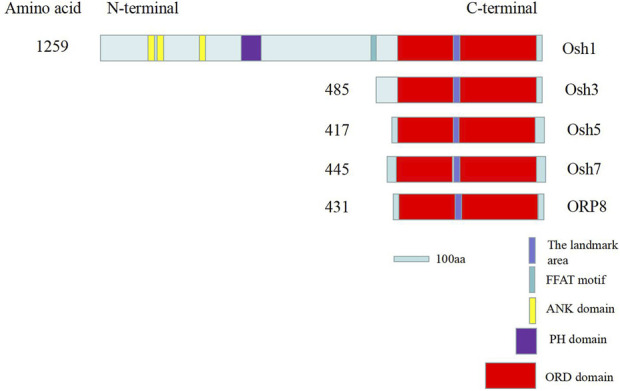
Schematic of key sequence domains of Osh proteins in *A. oryzae*. The “EQVSHHPP” sequence is the marker sequence of Osh proteins. ER was bound by FFAT sequences. Interactions between proteins and MCS are mediated by ANK domain (Kentala et al., 2015). PH domains interact with Golgi or PM by binding to the PIPs. Sterol and PIPs were mainly bound and exchanged by ORD domains between MCS (Ma et al., 2019).

The ORD domains of many ORP and Osh family proteins play an important role in the transport of sterols, and it contains a β-barrel domain *via* which sterols enter and are then transported to other organelles (Ma et al., 2019). In many species, “EQVSHHPP” is the signature of Osh proteins; however, it is “EKVSHRPV” in Osh3 and “EQTSHHPP” in Osh7 and ORP8. The reason behind this divergence remains unclear. Comparison of the *Osh* gene sequences of *A. nidulans* with the whole genome sequence of *A. oryzae* in the NCBI database revealed five *Osh* gene sequences in *A. oryzae*, which were named *Osh1* (XM_001825466.3), *Osh3* (XM_001824384.3), *Osh5* (XM_023235010.1), *Osh7* (XM_023237190.1), and *ORP8* (XM_001824352.3). Here, we studied only four Osh proteins (Osh3, Osh5, Osh7, and ORP8). Using the Smart online software, we found that Osh1 is a long chain protein with ORD domain, pleckstrin homology domain (PH domain), three ankyrin repeat domains (ANK), and two phenylalanines in an acidic tract (FFAT) motif. The other four Osh proteins are short-chain proteins with only ORD domains (Figure 5).

### Osh3 expression and purification

**FIGURE 6:**
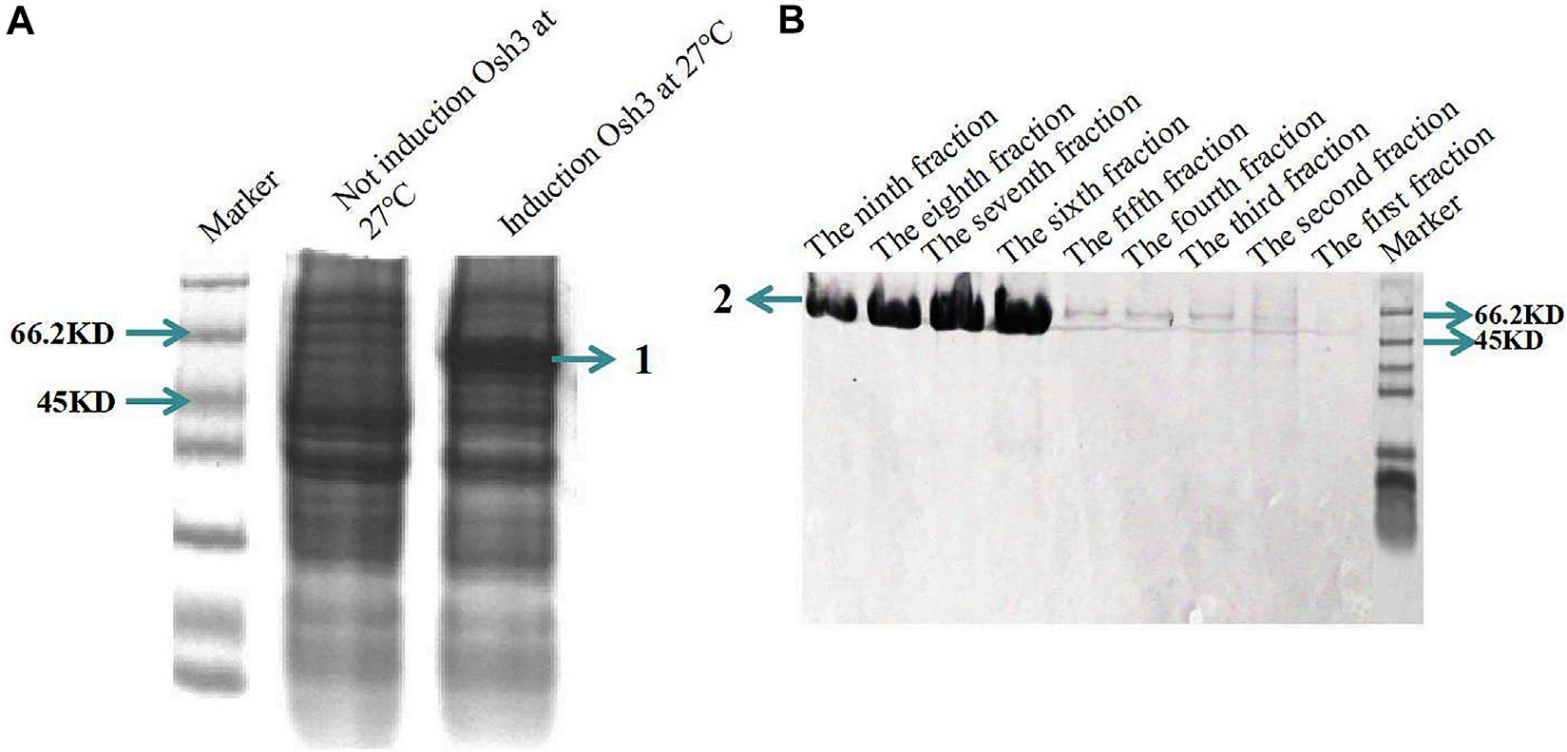
*Osh3* in pET28 vector was expressed in *E. coli Transseta* DE3 and purified using His Ni-agarose resin. **(A)** SDS-PAGE analysis of the induced Osh3. 1) Induced Osh3. **(B)** Osh3 was purified using His Ni-agarose resin; the amount of the purified protein was highest in fractions 6–9. Each fraction (tube) is 1 ml eluent. 2) Purified Osh3.

Osh3 was expressed in *E. coli Transseta* DE3 cells using 0.5 mM IPTG and cultured for 8 h at 27°C (Figure 6A). The size of Osh3 predicted by DNAMAN was 53.05 KD. As mentioned in the Methods section, the expressed protein was purified using the His Ni-agarose resin and buffers. The results showed that the yield of Osh3 was 1,056 μg/ml and large amounts of the purified protein were present in fractions 6–9 (Figure 6B). Non-specific proteins of molecular weight higher or lower than the expected weight were not observed in these fractions, indicating that the purified protein was highly pure. However, although the amount of the purified protein in fractions 3–5 was not as high (faint target band visible) as that in fractions 6–9, the purity of the target band was high. This may be due to the concentration gradient of the protein eluent. The imidazole concentration in the eluent in fractions 1–5 was 50 mM, while it was 100 mM in fractions 6–10. As the target protein was bound tightly to the His-Ni-agarose resin column, the low concentration of the elution buffer could not elute the target protein.

### Osh3 shows a binding preference for sterols

**FIGURE 7:**
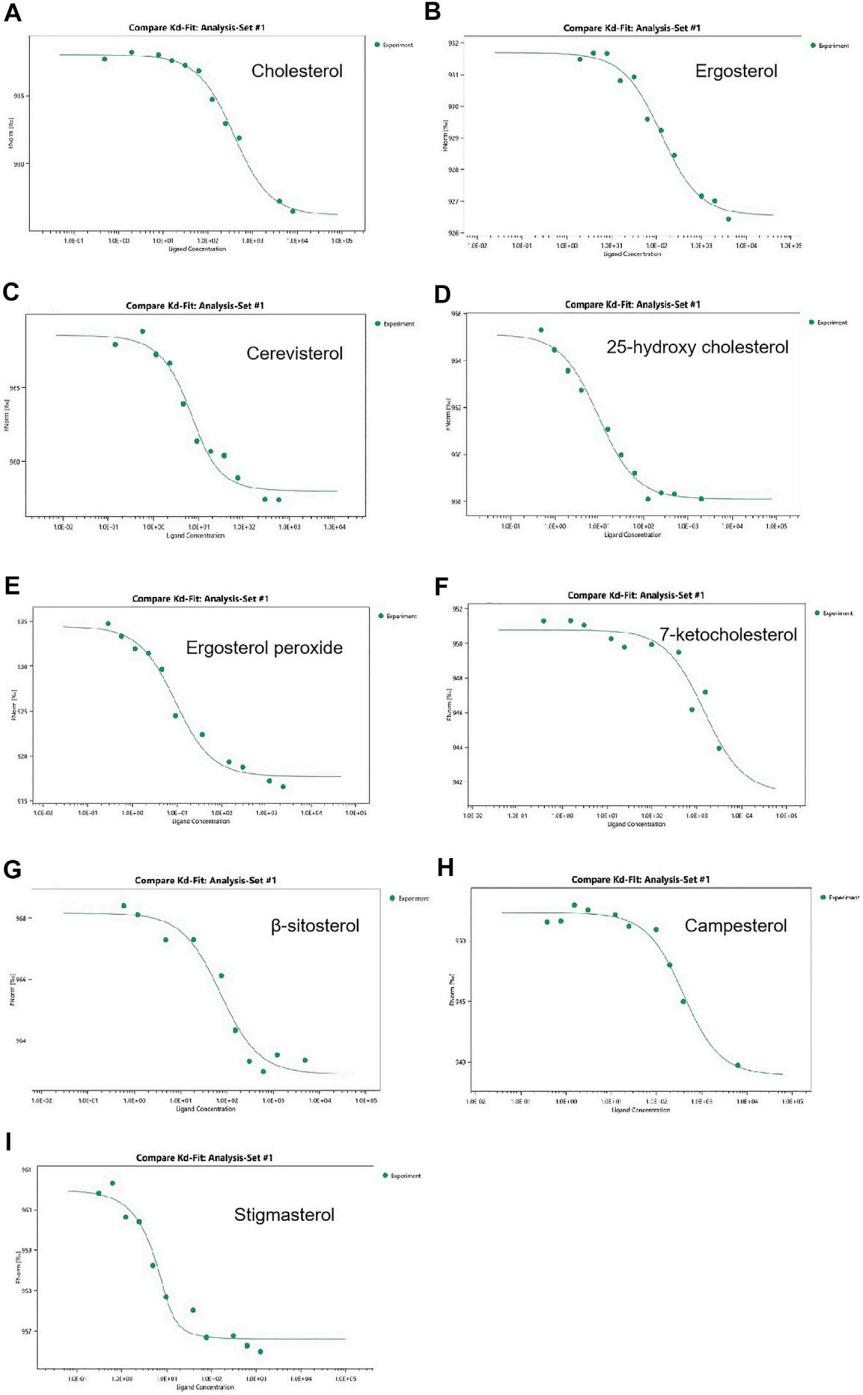
Determination of binding activity for ligand to Osh3 using the MST method. The binding affinity was analyzed using the NanoTemper monolith NT.115 instrument. **(A)** Kd of Osh3 for cholesterol was 332.9 ± 45.3 nM. **(B)** Kd of Osh3 for ergosterol was 121.9 ± 24.3 nM. **(C)** Kd of Osh3 for cerevisterol was 3.4 ± 1.3 nM. **(D)** Kd of Osh3 for 25-hydroxy cholesterol was 9.1 ± 2.0 nM. **(E)** Kd of Osh3 for ergosterol peroxide was 8.1 ± 2.1 nM. **(F)** Kd of Osh3 for 7-ketocholesterol was 1,455.6 ± 1,088.3 nM. **(G)** Kd of Osh3 for β-sitosterol was 65.8 ± 24.6 nM. **(H)** Kd of Osh3 for campesterol was 339.5 ± 78.1 nM. **(I)** Kd of Osh3 for stigmasterol was 1.0 ± 0.8 nM.

OSBP and ORPs bind various sterols (e.g., 25-hydroxycholesterol, 22-hydroxycholesterol, cholesterol, ergosterol) *via* the ORD domain and bind phospholipids *via* their PH domain (Kentala et al., 2015; Ma et al., 2019). OSBP and ORPs transport phospholipids formed on the plasma membrane or the membranes of other organelles to the ER, which are hydrolyzed by Sac1, and reverse transport sterols from the ER to the corresponding membranes for metabolic activities (Mesmin et al., 2017). All Osh proteins in *A. oryzae* possess ORD domains. To show that Osh proteins in *A. oryzae* perform functions similar to that of their homologs in the same family, we assessed the binding affinity of Osh3 with cholesterol, ergosterol, and other seven types of sterols using the MST assay. The Kd value reflects the binding ability between Osh3 and nine types of sterols. Binding affinity was higher when the Kd value was smaller. The results showed that Osh3 bound well with cholesterol, ergosterol, and the seven other sterols and exhibited a higher affinity to stigmasterol and cerevisterol (Figure 7). The Kd values of Osh3 with cholesterol, ergosterol and ergosterol peroxide were 332.9 ± 45.3 nM, 121.9 ± 24.3 nM, and 8.1 ± 2.1 nM, respectively (Figures 7A,B,E). This suggested that Osh3 can bind to sterols in cells and participate in the transport of cholesterol, ergosterol, and ergosterol peroxide potentially *via* the ORD domain, thereby transporting them to specific parts of cells to participate in intracellular lipid metabolism.

## Discussion

Here, we showed for the first time that *A. oryzae* can utilize cholesterol efficiently and instantaneously in the normal fermentation state. This indicated that *A. oryzae* possesses a mechanism of metabolizing cholesterol into other beneficial active substances, thereby promoting normal physiological function. However, the underlying metabolic mechanism remains unknown. Using cholesterol fermentation experiments with *A. oryzae*, we showed that the carbon source does not have to be dissolved in the medium before it can be utilized by *A. oryzae*, and that it can be absorbed and utilized by *A. oryzae* even when insoluble, which provides a good reference for the microbial fermentation experiment in this field. This study also provides a good reference for future research on microbial utilization of cholesterol. Cholesterol is an essential substance of the human body, accounting for 0.2% of the total body weight. Excessive cholesterol level leads to many diseases, which seriously threaten human’s health (Steinberg, 2006). Studies using radiolabeled cholesterol carbon atoms have shown that *A. niger* can absorb cholesterol, but the absorptivity is low, and the results is unclear (Nemec and Jernejc, 2002). *A. oryzae* is a filamentous fungus widely used in fermented food that does not produce mycotoxin. It is considered a safe food-grade fermentation strain (Sugiyama, 1984). It is listed as “generally recognized as safe (GRAS)” by the Food and Drug Administration (FDA) of the United States (Yohe et al., 2015). The results imply that *A. oryzae* not only produce flavor substances, but also reduce the cholesterol content in fermented food. This study is of considerable significance for the development of low cholesterol food and new drugs, which can be used to treat diseases caused by high cholesterol levels.

**FIGURE 8:**
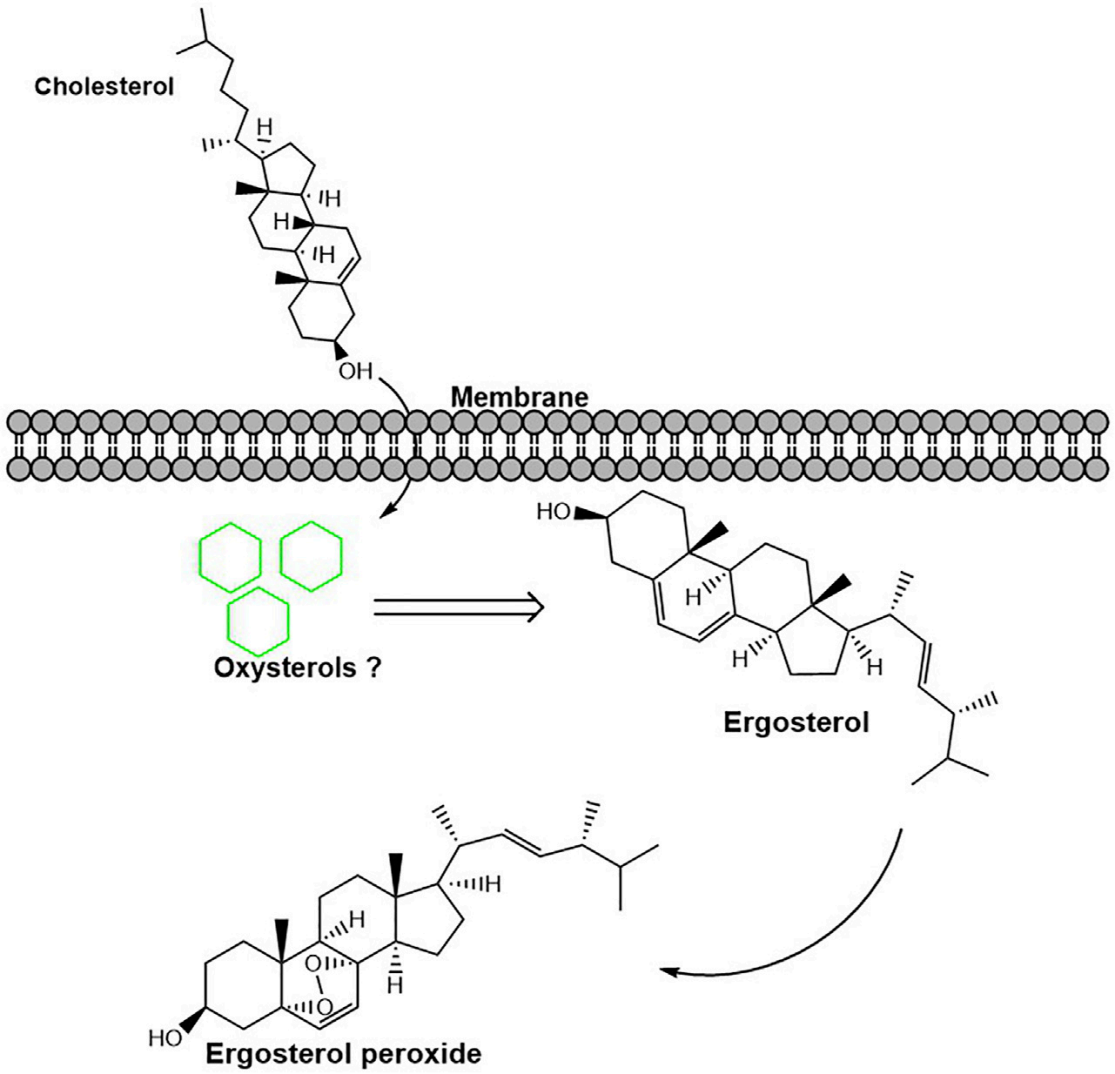
Cholesterol utilization by *A. oryzae* can promote the conversion of ergosterol to ergosterol peroxide. After cholesterol was utilized by *A. oryzae*, some cholesterol was converted into other metabolites. This process promotes the conversion of ergosterol to ergosterol peroxide.

Our results showed that *A. oryzae* can synthesize ergosterol peroxide for the first time and that instantaneous cholesterol utilization by *A. oryzae* can promote the rapid synthesis of ergosterol peroxide. At the same time, cholesterol utilization by *A. oryzae* reduced the ergosterol content in *A. oryzae*. Some studies have shown that ergosterol peroxide is the oxide of ergosterol (Wei et al., 2009). It is speculated that the reduction in ergosterol level is likely because of its conversion into ergosterol peroxide. *A. oryzae* possesses a mechanism to regulate the conversion of ergosterol to ergosterol peroxide, and cholesterol utilization can promote this process (Figure 8). The content of ergosterol is relatively stable in *A. oryzae*; however, this study demonstrated that the utilization of cholesterol by *A. oryzae* reduced the ergosterol content in cells. Cholesterol can readily insert into lipid membranes and impact their physical properties (Antonny et al., 2018). Therefore, we speculated that a part of the cholesterol utilized by *A. oryzae* is metabolized, whereas the rest may supplement ergosterol’s cellular functions, such as providing a substitute for membrane ergosterol (Xiong et al., 2005). This suggests that when cholesterol is the carbon source, the Osh proteins expressed by the *Osh* genes simultaneously transport cholesterol, ergosterol, and ergosterol peroxide to other cellular localization for metabolism. In contrast, when glucose is the carbon source, the Osh proteins only transport ergosterol and ergosterol peroxide as transport substances for cellular metabolism. Some research reported that oxysterols are by-products of cholesterol that form spontaneously or enzymatically, so we speculate that a part of cholesterol may be converted into ergosterol peroxide and other oxysterols (Luu et al., 2016). However, these are still unclear and warrant further investigations. Ergosterol peroxide is a bioactive molecule with high anticancer, antitumor, and antituberculous activity produced by mushrooms (Takei et al., 2005). However, the ergosterol peroxide content in mushrooms is low (0.1%–0.29%) (Krzyczkowski et al., 2009). In addition, mushroom fungi need a few months to grow as they have a long growth cycle. This study showed for the first time that *A. oryzae* can not only produce flavor substances but can also produce ergosterol peroxide, which has medicinal value; in addition, the ergosterol peroxide content in *A. oryzae* is significantly higher than that in other fungi. *A. oryzae* can promote the production of this active substance after utilizing cholesterol in the environment. The ergosterol peroxide content in *A. oryzae* reached 0.712% after culturing in the cholesterol medium for 48 h. Thus, this study is essential for developing new anti-cancer and anti-tuberculosis drugs.

Recent studies have shown that Osh1 in *A. oryzae* can bind to oxysterols and that its binding affinity differs with different oxysterols; the Kd values for the binding of Osh1 to β-sitosterol, stigmasterol, 25-hydroxycholesterol, and 7-Ketocholesterol were 81.7 ± 7 nM, 115 ± 11 nM, 21.1 ± 3 nM, and 25.4 ± 4 nM respectively (Ma et al., 2019). Osh proteins are important proteins involved in sterols metabolism in eukaryotic organisms and play essential roles in sterols transport (Antonny et al., 2018). The sterols synthesized on the ER are transported to other organelles for metabolic degradation, and the lipids synthesized on other organelles are transported to the ER for metabolic reaction (Pietrangelo and Ridgway, 2018). Research showed that OSBP could change its cellular localization rapidly upon adding oxysterol to promote sphingomyelin synthesis (Lagace et al., 1999; Antonny et al., 2018). So we can speculate that Osh proteins in *A. oryzae* change their location quickly after cholesterol addition to promote the transportation and metabolism of sterols. Recent studies have shown that ORP2 in the human body can transport sterols and participate in the regulation of AKT signaling pathway, thereby playing an important role in glycolysis and glycogen synthesis (Kentala et al., 2018). Currently, OSBP and Osh proteins are being studied extensively worldwide, especially the functions of Osh proteins in *S. cerevisiae, Candida albicans,* and *Aspergillus nestoris*. However, studies on Osh proteins in *A. oryzae* are limited (Qiu and Zeng, 2019). Our study showed that *A. oryzae* harbors five Osh family proteins, among which Osh1 has been reported previously (Ma et al., 2019). Osh5, Osh7, and ORP8 can be induced for expression, although the yield after purification is too low, and the conditions still require optimization. Hence, we only discussed research relevant to Osh3 in this article. We showed that Osh3 has high binding affinity for cholesterol, ergosterol, ergosterol peroxide, and other six types of sterols, demonstrating that the functions of *A. oryzae* Osh3 were similar to those of other Osh proteins in its family (Wang et al., 2019). This suggested that Osh3 is involved in lipid metabolism and regulation in *A. oryzae* and plays an important role in sterol (e.g., cholesterol, ergosterol, and ergosterol peroxide) transport and the conversion of ergosterol to ergosterol peroxide.

## Conclusion

Based on these findings, we concluded that *A. oryzae* could absorb and utilize cholesterol instantaneously and efficiently. Hence, the application of *A. oryzae* in the production of cholesterol-lowering food and feed and the development of cholesterol-lowering drugs shows tremendous prospect. However, more exploration is needed to achieve this goal. At the same time, *A. oryzae* can synthesize ergosterol peroxide in large quantities, which may provide a good reference for the development of anticancer, antitumor, and anti-tuberculous drugs.

## Data Availability

The original contributions presented in the study are included in the article/supplementary material, further inquiries can be directed to the corresponding author.

## References

[B1] Antonny B. Bigay J. Mesmin B. (2018). The oxysterol-binding protein cycle: Burning off PI(4)P to transport cholesterol. Annu. Rev. Biochem. 87, 809–837. 10.1146/annurev-biochem-061516-044924 29596003

[B2] Bechman A. Phillips R. D. Chen J. (2012). Changes in selected physical property and enzyme activity of rice and barley koji during fermentation and storage. J. Food Sci. 77, M318–M322. 10.1111/j.1750-3841.2012.02691.x 22583119

[B3] Bühler N. Hagiwara D. Takeshita N. (2015). Functional analysis of sterol transporter orthologues in the filamentous fungus aspergillus nidulans. Eukaryot. Cell 14, 908–921. 10.1128/EC.00027-15 26116213 PMC4551590

[B4] Damodharan K. Palaniyandi S. A. Yang S. H. Suh J. W. (2016). Functional probiotic characterization and *in vivo* cholesterol-lowering activity of Lactobacillus helveticus isolated from fermented cow milk. J. Microbiol. Biotechnol. 26, 1675–1686. 10.4014/jmb.1603.03005 27435541

[B5] Ding W. Shi C. Chen M. Zhou J. Long R. Guo X. (2017). Screening for lactic acid bacteria in traditional fermented Tibetan yak milk and evaluating their probiotic and cholesterol-lowering potentials in rats fed a high-cholesterol diet. J. Funct. Foods 32, 324–332. 10.1016/j.jff.2017.03.021

[B6] Du X. Brown A. J. Yang H. (2015). Novel mechanisms of intracellular cholesterol transport: Oxysterol-binding proteins and membrane contact sites. Curr. Opin. Cell Biol. 35, 37–42. 10.1016/j.ceb.2015.04.002 25932595

[B7] Dupont S. Lemetais G. Ferreira T. Cayot P. Gervais P. Beney L. (2012). Ergosterol biosynthesis: a fungal pathway for life on land? Evolution 66, 2961–2968. 10.1111/j.1558-5646.2012.01667.x 22946816

[B8] Kentala H. Koponen A. Vihinen H. Pirhonen J. Liebisch G. Pataj Z. (2018). OSBP-related protein-2 (ORP2): a novel akt effector that controls cellular energy metabolism. Cell. Mol. Life Sci. 75, 4041–4057. 10.1007/s00018-018-2850-8 29947926 PMC11105326

[B9] Kentala H. Pfisterer S. G. Olkkonen V. M. Weber-Boyvat M. (2015). Sterol liganding of OSBP-related proteins (ORPs) regulates the subcellular distribution of ORP-VAPA complexes and their impacts on organelle structure. Steroids 99, 248–258. 10.1016/j.steroids.2015.01.027 25681634

[B10] Krzyczkowski W. Malinowska E. Suchocki P. Kleps J. Olejnik M. Herold F. (2009). Isolation and quantitative determination of ergosterol peroxide in various edible mushroom species. Food Chem. 113, 351–355. 10.1016/j.foodchem.2008.06.075

[B11] Lagace T. A. Byers D. M. Cook H. W. Ridgway N. D. (1999). Chinese hamster ovary cells overexpressing the oxysterol binding protein (OSBP) display enhanced synthesis of sphingomyelin in response to 25-hydroxycholesterol. J. Lipid Res. 40, 109–116. 10.1016/s0022-2275(20)33345-9 9869656

[B12] Li Y. Zhang H. Fan J. Chen Z. Chen T. Zeng B. (2021). A highly efficient identification of mutants generated by CRISPR/Cas9 using the non-functional DsRed assisted selection in Aspergillus oryzae. World J. Microbiol. Biotechnol. 37, 132. 10.1007/s11274-021-03100-8 34240255

[B13] Luu W. Sharpe L. J. Capell-Hattam I. Gelissen I. C. Brown A. J. (2016). Oxysterols: Old tale, new twists. Annu. Rev. Pharmacol. Toxicol. 56, 447–467. 10.1146/annurev-pharmtox-010715-103233 26738477

[B14] Ma L. Zhang X. Hu Z. He B. Ai M. Zeng B. (2019). Heterologous expression and functional characterization of the ligand-binding domain of oxysterol-binding protein from Aspergillus oryzae. Braz. J. Microbiol. 50, 415–424. 10.1007/s42770-019-00060-y 30848436 PMC6863284

[B15] Mesmin B. Bigay J. Polidori J. Jamecna D. Lacas-Gervais S. Antonny B. (2017). Sterol transfer, PI4P consumption, and control of membrane lipid order by endogenous OSBP. Embo J. 36, 3156–3174. 10.15252/embj.201796687 28978670 PMC5666618

[B16] Nemec T. Jernejc K. (2002). Influence of Tween 80 on lipid metabolism of an Aspergillus niger strain. Appl. Biochem. Biotechnol. 101, 229–238. 10.1385/abab:101:3:229 12109818

[B17] Niu T. G. (2000). Mechanism and application of microbial bioconversion of cholesterol in food. Beijing: China Agricultural University. PhD.

[B18] Pietrangelo A. Ridgway N. D. (2018). Bridging the molecular and biological functions of the oxysterol-binding protein family. Cell. Mol. Life Sci. 75, 3079–3098. 10.1007/s00018-018-2795-y 29536114 PMC11105248

[B19] Qiu S. Zeng B. (2019). Advances in understanding of the oxysterol-binding protein homologous in yeast and filamentous fungi. Int. Microbiol. 22, 169–179. 10.1007/s10123-019-00056-6 30810998

[B20] Solanko L. M. Sullivan D. P. Sere Y. Y. Szomek M. Lunding A. Solanko K. A. (2018). Ergosterol is mainly located in the cytoplasmic leaflet of the yeast plasma membrane. Traffic 19, 198–214. 10.1111/tra.12545 29282820

[B21] Steinberg D. (2006). Thematic review series: the pathogenesis of atherosclerosis. An interpretive history of the cholesterol controversy, part V: the discovery of the statins and the end of the controversy. J. Lipid Res. 47, 1339–1351. 10.1194/jlr.R600009-JLR200 16585781

[B22] Sugiyama S.-I. (1984). Selection of micro-organisms for use in the fermentation of soy sauce. Food Microbiol. 1, 339–347. 10.1016/0740-0020(84)90067-4

[B23] Takei T. Yoshida M. Ohnishi-Kameyama M. Kobori M. (2005). Ergosterol peroxide, an apoptosis-inducing component isolated from Sarcodon aspratus (Berk.) S. Ito. Biosci. Biotechnol. Biochem. 69, 212–215. 10.1271/bbb.69.212 15665489

[B24] Taylor F. R. Kandutsch A. A. (1985). Oxysterol binding protein. Chem. Phys. Lipids 38, 187–194. 10.1016/0009-3084(85)90066-0 4064220

[B25] Wang H. Ma Q. Qi Y. Dong J. Du X. Rae J. (2019). ORP2 delivers cholesterol to the plasma membrane in exchange for phosphatidylinositol 4, 5-bisphosphate (PI(4, 5)P(2)). Mol. Cell 73, 458–473. 10.1016/j.molcel.2018.11.014 30581148

[B26] Wang Q. G. Xiao X. Li Q. Ma X. (2018). Determination of cholesterol content in various dairy products with high performance liquid chromatography. Dis. Prev. Control Bull. 33, 81–83. 10.13215/j.cnki.jbyfkztb.1807005

[B27] Wei D. D. Xu Q. M. Sun X. F. (2009). Structure modification of ergosterin in Bulgaria inquinants and its antituberculous activity. Lishizhen Med. Mater. Med. Res. 20, 2312–2314. 10.3969/j.issn.1008-0805.2009.09.101

[B28] Xiong Q. Hassan S. A. Wilson W. K. Han X. Y. May G. S. Tarrand J. J. (2005). Cholesterol import by Aspergillus fumigatus and its influence on antifungal potency of sterol biosynthesis inhibitors. Antimicrob. Agents Chemother. 49, 518–524. 10.1128/AAC.49.2.518-524.2005 15673727 PMC547240

[B29] Yohe T. T. O'diam K. M. Daniels K. M. (2015). Growth, ruminal measurements, and health characteristics of Holstein bull calves fed an Aspergillus oryzae fermentation extract. J. Dairy Sci. 98, 6163–6175. 10.3168/jds.2015-9313 26142841

[B30] Yu S. Cui H. (2001). Advances in the healthy foods of China. Beijing: People's Medical Publishing House.

[B31] Zhou G. Chen Y. Kong Q. Ma Y. Liu Y. (2017). Detoxification of aflatoxin B_1_ by zygosaccharomyces rouxii with solid state fermentation in peanut meal. Toxins (Basel) 9, E42. 10.3390/toxins9010042 28117705 PMC5308274

